# 2-Hydroxylation is a chemical switch linking fatty acids to glucose-stimulated insulin secretion

**DOI:** 10.1016/j.jbc.2024.107912

**Published:** 2024-10-21

**Authors:** Hong Li, Lin Lin, Xiaoheng Huang, Yang Lu, Xiong Su

**Affiliations:** 1 School of Life Sciences, Suzhou Medical College of Soochow University, Suzhou, Jiangsu, China; 2 MOE Key Laboratory of Geriatric Diseases and Immunology, Suzhou Medical College of Soochow University, Suzhou, Jiangsu, China; 3 Suzhou Key Laboratory of Systems Biomedicine, Suzhou Medical College of Soochow University, Suzhou, Jiangsu, China

**Keywords:** β-cells, diabetes, fatty acid, FA2H, fatty acid 2-hydroxylation, glucose transport, GLUT2, insulin secretion, metabolism

## Abstract

Glucose-stimulated insulin secretion (GSIS) in pancreatic β-cells is metabolically regulated and progressively diminished during the development of type 2 diabetes (T2D). This dynamic process is tightly coupled with fatty acid metabolism, but the underlying mechanisms remain poorly understood. Fatty acid 2-hydroxylase (FA2H) catalyzes the conversion of fatty acids to chiral specific (*R*)-2-hydroxy fatty acids ((*R*)-2-OHFAs), which influences cell metabolism. However, little is known about its potential coupling with GSIS in pancreatic β cells. Here, we showed that *Fa2h* knockout decreases plasma membrane localization and protein level of glucose transporter 2 (GLUT2), which is essential for GSIS, thereby controlling blood glucose homeostasis. Conversely, FA2H overexpression increases GLUT2 on the plasma membrane and enhances GSIS. Mechanistically, FA2H suppresses the internalization and trafficking of GLUT2 to the lysosomes for degradation. Overexpression of wild-type FA2H, but not its mutant with impaired hydroxylase activity in the pancreatic β-cells, improves glucose tolerance by promoting insulin secretion. Levels of 2-OHFAs and *Fa2h* gene expression are lower in high-fat diet-induced obese mouse islets with impaired GSIS. Moreover, lower gene expression of *FA2H* is observed in a set of human T2D islets when the insulin secretion index is significantly suppressed, indicating the potential involvement of FA2H in regulating mouse and human GSIS. Collectively, our results identified an FA chemical switch to maintain the proper response of GSIS in pancreatic β cells and provided a new perspective on the β-cell failure that triggers T2D.

Type 2 diabetes (T2D) is a multifactorial disease characterized by decreased peripheral glucose uptake, increased hepatic gluconeogenesis, and impaired β-cell functions (1). A progressive decrease in insulin secretory capacity has been identified as a critical factor contributing to the development of T2D (2, 3). Glucose, free fatty acids, and amino acids serve as nutrient stimuli for insulin secretion through their sensing, production of key signaling metabolites, and metabolism to generate ATP (4). Glucose-stimulated insulin secretion (GSIS) represents the primary mechanism of circulating insulin regulation to maintain glucose homeostasis. In pancreatic β cells, glucose is taken up through glucose transporters (GLUTs) and metabolized to produce ATP, which prompts the closure of ATP-sensitive K^+^ channels (K_ATP_), evoking membrane depolarization and subsequent opening of voltage-gated Ca^2+^ channels. The resulting calcium influx drives insulin granules' exocytosis (5). An increase in ATP alone is insufficient for GSIS, and additional metabolic coupling factors (MCFs) and the K_ATP_ channel-independent amplifying actions of glucose also influence insulin secretion (6).

Fatty acid (FA) signaling is generally regarded as a critical and possibly pathogenic factor in controlling GSIS (7), but the precise pathways and molecules involved in the process remain elusive. The acute rise in free fatty acids (FFAs) potently augments GSIS partially by the direct activation of the cell surface FFA1/GPR40 receptor (FFAR1) and intracellular metabolism to generate MCFs (4, 8). On the other hand, the sustained positive influx of FAs due to the persistently elevated rate of lipolysis in adipose tissue with insulin resistance is considered a precipitating factor for β-cell dysfunction and impaired GSIS (7). Thus, knowledge regarding islet FA metabolism is critically important to understand the complex interplay between the nature of the elevated plasma lipids and glucose dynamics in obesity.

The diversity of FA structures is manifested by backbone branching, desaturation, and chemical modification, which influence their functions in membrane properties and cellular signaling. Indeed, the insulinotropic effects of individual FAs are markedly influenced by chain length and degree of unsaturation (9). FA derivatives by hydroxylation naturally occur in mammalian cell lipids, which can be generated by various hydroxylases or provided by microorganisms or food intake (10, 11). However, the potential coupling of FA hydroxylation with GSIS has not been investigated. Fatty acid 2-hydroxylase (FA2H) generates (*R*)-2-hydroxy FAs ((*R*)-2-OHFAs), which are incorporated into complex lipids or degraded by α oxidation (12). FA2H has been associated with leukodystrophy and spastic paraparesis in humans and is essential for the permeability barrier function in the epidermis (13). The physiological functions of FA2H in other organs remain largely unknown. Our previous studies have revealed that FA2H is related to glucose utilization and metabolic processes in the stomach and colorectal cancers (14, 15) and in adipocytes (16), suggesting the presence of an unexplored linkage between FA 2-hydroxylation and glucose metabolism in the regulation of β cell GSIS. Importantly, a recent study using the collaborative cross-mouse reference population to identify novel quantitative trait loci and their diabetogenic effects in response to high-fat dietary (HFD) revealed that the *Fa2h* gene may be associated with host glucose tolerance (17).

There are 14 facilitative diffusion GLUTs, and GLUT2 is well established as the principal membrane GLUT with low affinity in rodent pancreatic β-cells. Loss of β-cell GLUT2 expression is associated with hyperglycemia and diminished GSIS, which could be restored by pancreatic-specific expression of GLUT2 (18). Glucose transport affects the maximal rates of GSIS, while its subsequent phosphorylation by glucokinase decreases the half-maximal effective concentration (EC50) values for glucose (19). Surface expression of β-cell GLUT2 is essential for GSIS, and GLUT2 glycosylation mediates a link between diet and insulin production (20). Altered GLUT2 protein level, trafficking, or plasma membrane localization were subsequently reported in numerous studies on GSIS regulated by WASH (Wiskott-Aldrich syndrome protein and SCAR homolog) (21), the zinc transporter Slc39a5 (22), Ras homolog enriched in brain 1 (Rheb1) (23), ghrelin receptor (24), and 17β-estradiol (25).

FA2H in the pancreas is mainly expressed in endocrine cells, but its function in β cells and potential association with the development of diabetes has not been explored. In this study, we identified an essential role of FA2H in regulating GLUT2 internalization and trafficking to lysosomes for degradation, leading to altered GSIS. The dependency of FA2H function on 2-hydroxylation activity was confirmed by mutation with impaired hydroxylation activity and supplementation of its enzymatic product. We discovered a physiological role of FA2H in GSIS, which represents a chemical linkage between FA metabolism and insulin secretion in pancreatic β cells.

## Results

### FA2H regulates glucose-stimulated insulin secretion

**Figure 1:**
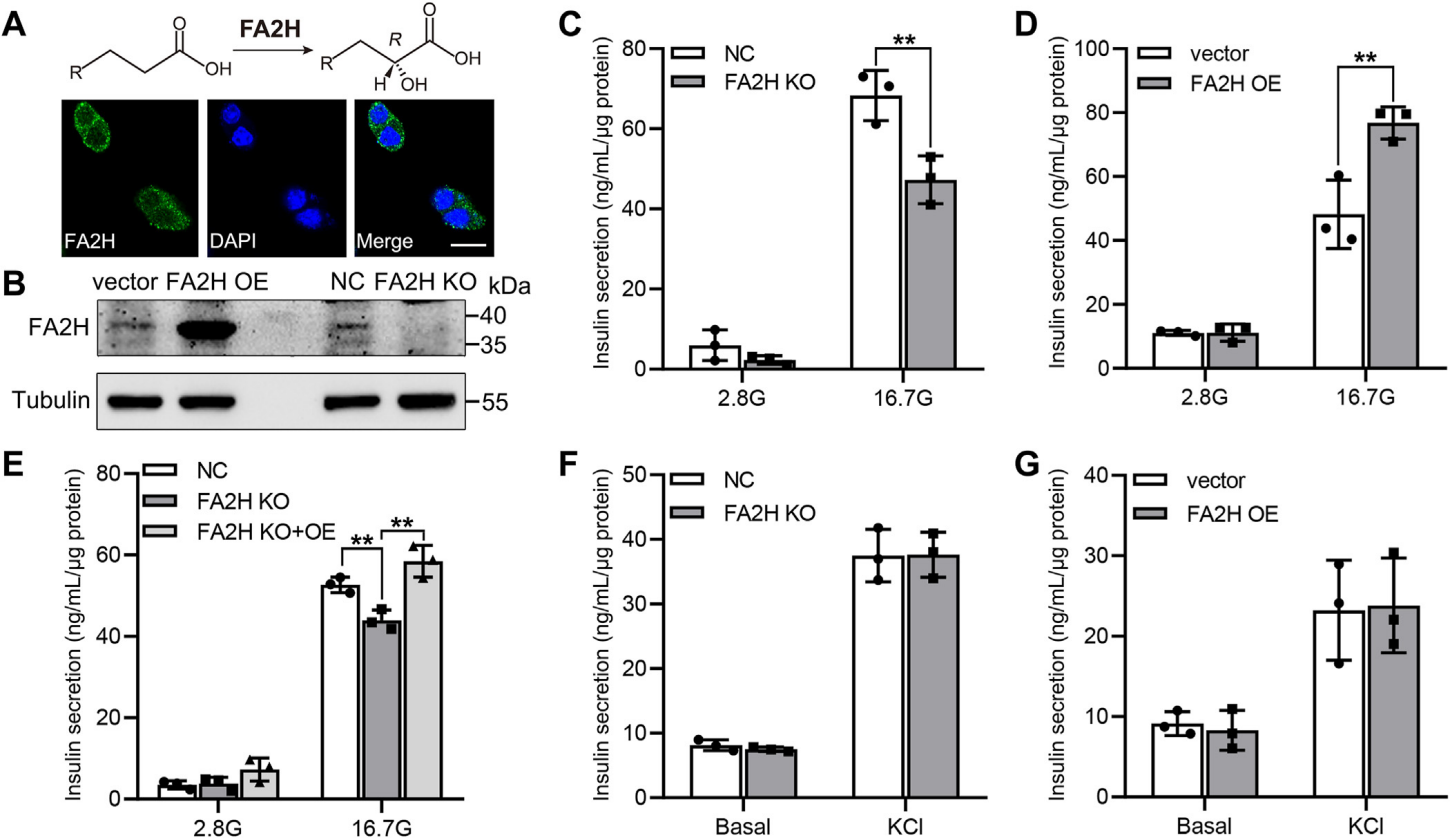
**FA2H regulates GSIS but not KSIS in MIN6 cells.***A*, immunofluorescence staining of FA2H in MIN6 cells. Scale bar, 20 μm. *B*, whole-cell lysates of negative control (NC) cells, cells with FA2H knockout (KO), cells transfected with empty vector (vector), and cells stably over-expressing FA2H (OE) were analyzed by Western blotting using antibodies as indicated. *C*, GSIS (at low [2.8 mM, 2.8G] and high [16.7 mM, 16.7G] glucose conditions) in NC cells or cells with FA2H KO. *D*, GSIS in vector cells or cells with FA2H OE. *E*, GSIS in NC cells, FA2H KO cells, and FA2H KO cells reexpressing FA2H (KO + OE). F, KSIS in NC cells or cells with FA2H KO. *G*, KSIS in vector cells or cells with FA2H OE. Values represent the mean ± SD of three independent biological experiments∗∗, *p* < 0.01 by two-way ANOVA.

We examined FA2H expression in the glucose-responsive insulinoma MIN6 cells by immunofluorescence staining and observed strong immunoreactivity (Fig. 1*A*). Its the endoplasmic reticulum (ER) localization was confirmed by co-localization of FA2H-EGFP with the ER marker GRP94 (Fig. S1*A*). To explore the role of FA2H in β cell insulin secretion, we generated FA2H knockout (FA2H KO) MIN6 cells by CRISPR/Cas9 and FA2H overexpression (FA2H OE) cells by lentivirus infection (Fig. 1*B*). Under the basal condition of 2.8 mM glucose, FA2H KO or OE has minimal effects on insulin secretion. Treatment with 16.7 mM glucose dramatically stimulates insulin secretion, which is significantly suppressed by FA2H KO while being enhanced by FA2H OE (Fig. 1, *C* and *D*). Moreover, the re-expression of FA2H in FA2H KO cells can reverse the suppressed GSIS (Fig. 1*E*). We next examined FA2H regulation of insulin secretion in response to high KCl and found that FA2H KO or OE has no effects on KCl-stimulated insulin secretion (KSIS) (Fig. 1, *F* and *G*), suggesting that FA2H does not function through K_ATP_-independent pathways which are primarily responsible for the second phase of GSIS. Analysis of intracellular insulin content by ELISA or immunofluorescence staining revealed no significant difference in insulin content in cells with FA2H KO (Fig. S1, *B* and *C*) or OE (Fig. S1, *D* and *E*). Neither FA2H KO nor FA2H OE affects the mRNA levels of the critical genes in insulin synthesis, including pancreatic duodenal homeobox 1 (*Pdx*1), forkhead box protein O1 (*FoxO1*), and insulin 2 (*Ins2*) (Fig. S1, *F* and *G*).

### FA2H does not affect cytoskeleton, ER-mitochondria association integrity, or ER stress signaling pathways

**Figure 2:**
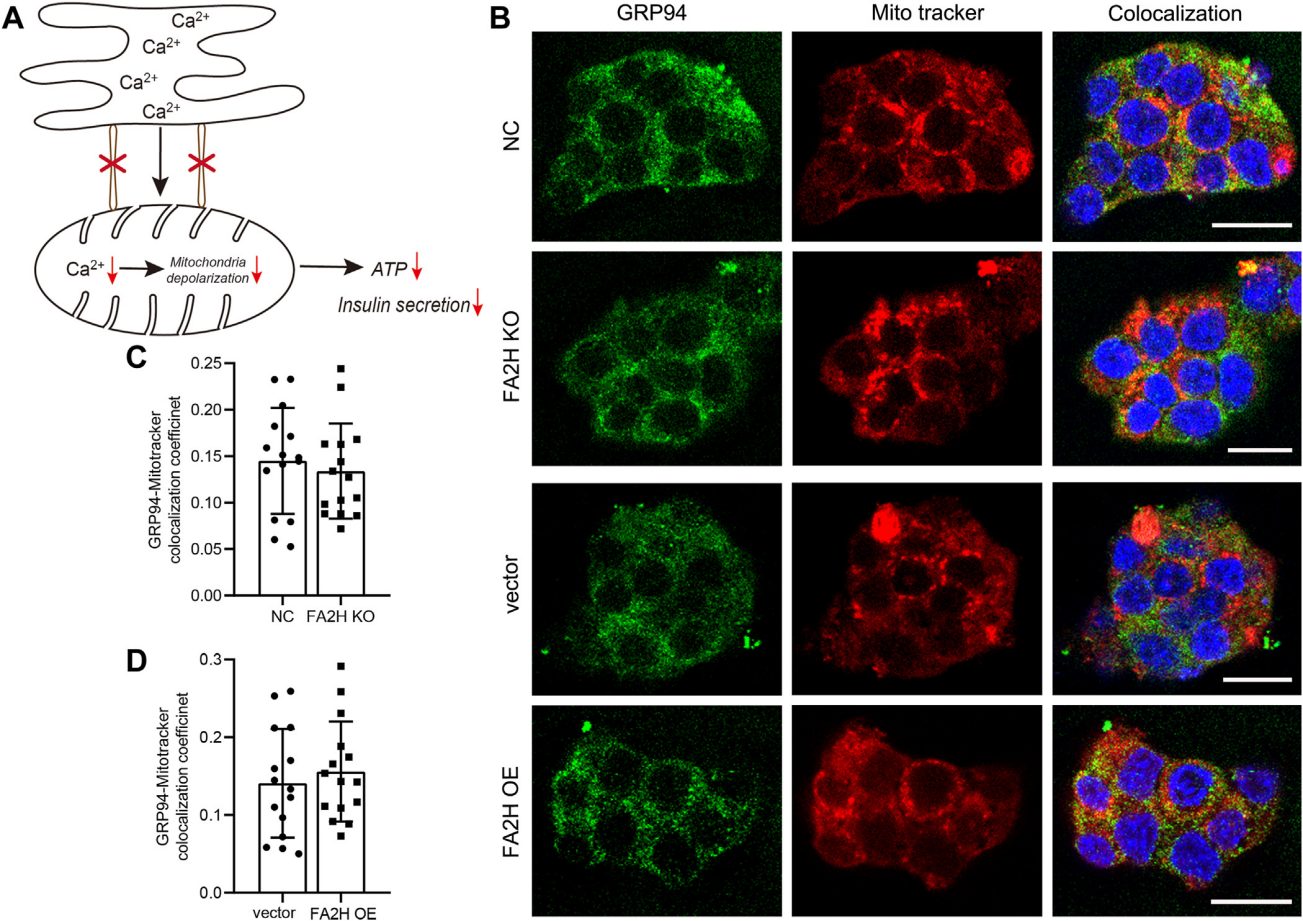
**FA2H does not affect the ER-mitochondria contact.***A*, ER-mitochondrial association regulates insulin secretion. *B*, the co-localization of ER marker GPR94 and Mito tracker negative control (NC) cells, cells with FA2H knockout (KO), cells transfected with empty vector (vector), and cells stably over-expressing FA2H (OE). Scale bar, 10 μm. *C* and *D*, pearson correlation coefficient analysis of (*B*). Values represent mean ± SD, n = 15 cell clusters.

The delivery of secretory granules is random Brownian motion rather than directional motion in β cells, and the cytoskeleton restricts the movement of secretory granules and insulin secretion (26). However, FA2H KO or OE does not affect the organization of microfilaments and microtubule networks (Fig. S2, *A* and *B*). In pancreatic islets, mitochondrial metabolism regulates insulin release by increasing the ATP/ADP ratio, and the ER controls insulin synthesis, correct folding, and sorting (27). ER-mitochondria interaction controls Ca^2+^ dynamics by regulating Ca^2+^ exchange, which plays a crucial function in GSIS (Fig. 2*A*) (28). However, FA2H KO or OE does not affect the co-localization of ER marker GRP94 with MitoTracker (Fig. 2*B*), and the co-localization coefficients of the two organelles are not significantly changed in FA2H KO or OE cells in comparison with their control cells (Fig. 2, *C* and *D*). These results demonstrate that FA2H does not regulate GSIS *via* an altered cytoskeleton or the integrity of the ER-mitochondria association. Given that prolonged ER stress can impair β cell function, we investigated whether FA2H KO or OE influences ER stress. Our analysis revealed no changes in ER stress-related signaling pathways (Fig. S3, *A*–*D*), suggesting that ER stress is unlikely to be involved in FA2H-mediated regulation of GSIS.

### FA2H deficiency promotes GLUT2 internalization and trafficking to lysosomes in β cells

**Figure 3:**
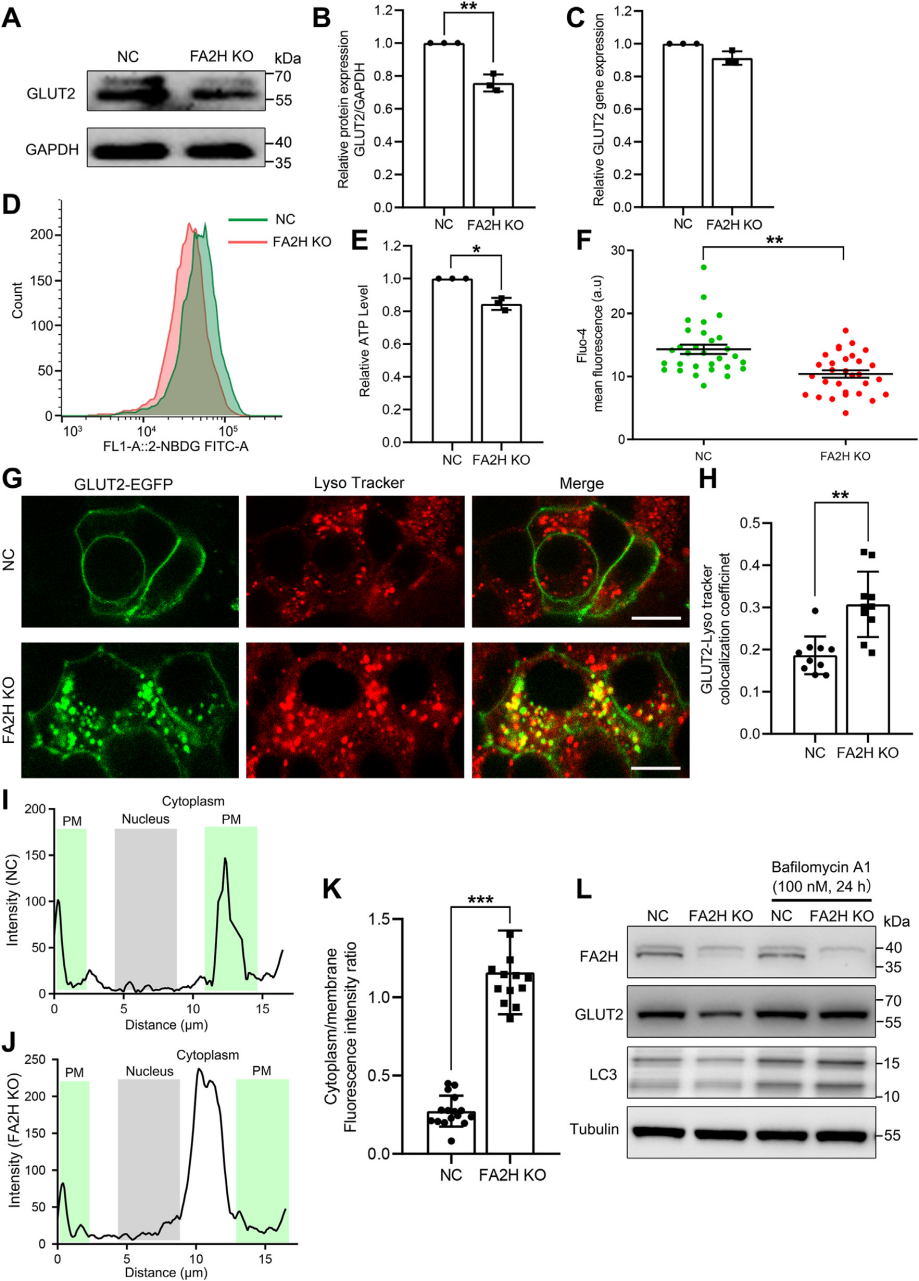
**FA2H deficiency promotes lysosomal degradation of GLUT2.***A* and *B*, whole-cell lysates of negative control (NC) MIN6 cells and cells with FA2H knockout (KO) were analyzed by Western blotting using antibodies as indicated and the band intensities of three independent experiments were quantified by ImageJ. *C*, qRT-PCR analyses of the mRNA samples prepared from NC and FA2H KO cells (n = 3 biological experiments). *D*, NC and FA2H KO MIN6 cells were incubated with 30 μmol/L fluorescent glucose analog 2-NBDG, and its uptake was assayed by flow cytometry. *E*, intracellular ATP levels in NC and FA2H KO cells (n = 3 biological experiments). *F*, intracellular Ca^2+^ levels were measured with the Ca^2+^-dependent fluorescent dye Fluo-4 (n = 30 cell clusters). G, NC and FA2H KO cells were transfected with GLUT2-GFP plasmid (*green*) and stained with Lysotracker (*red*). Scale bar, 10 μm. *H*, pearson correlation coefficients of GFP with Lyso tracker from NC and FA2H KO cells (n = 10 cell clusters). *I* and *J*, distribution of GLUT2-EGFP in the negative control (NC) and cells with FA2H knockout (KO) MIN6 cells. *K*, relative membrane and cytoplasm fluorescent intensity ratio of GFP in NC or FA2H KO cells were quantified by ImageJ (n = 10 cell clusters). *L*, FA2H KO or control (NC) MIN6 cells were treated with 100 nM Bafilomycin A1 for 24 h and the whole-cell lysates were examined by Western blotting using antibodies as indicated. Values represent mean ± SD, ∗, *p* < 0.05, ∗∗, *p* < 0.01, ∗∗∗, *p* < 0.001 by Student’s *t* test.

GLUT2 is the most abundant GLUT isoform in rodent β cells, which is required for GSIS (29). We investigated the potential regulation of GLUT2 protein by FA2H in MIN6 cells and found that its level is significantly reduced in FA2H KO cells compared to control cells (Fig. 3, *A* and *B*). In contrast, its mRNA level is not affected (Fig. 3*C*). Consistently, FA2H KO cells have a decreased uptake of 2-(N-(7-nitrobenz-2-oxa-1,3-diazol-4-yl) amino)-2-deoxyglucose (2-NBDG), a fluorescent analog of glucose (Fig. 3*D*). Moreover, the ATP and Ca^2+^ levels are also significantly reduced in the absence of FA2H (Fig. 3, *E* and *F*). These results indicate that FA2H deletion decreases overall GLUT2 protein levels and limits glucose utilization in MIN6 cells. In pancreatic β-cells, GLUT2 undergoes plasma membrane endocytosis and lysosomal degradation in response to glucose (30). FA2H knockout significantly increases the intracellular localization of GLUT2-EGFP (Fig. 3, *G* and *H*). Moreover, the co-localization of GLUT2-EGFP with mCherry-tagged caveolin 1 in FA2H KO cells is increased substantially compared to control cells (Fig. S4, *A* and *B*). In contrast, its co-localization with the mCherry-Clathrin light chain is not affected (Fig. S4, *C* and *D*), suggesting that FA2H specifically influences caveolae-dependent endocytosis and trafficking. Our previous study showed that FA2H knockdown enhances the lysosomal accumulation of GLUT4 in adipocytes (16). We examined FA2H regulation of the subcellular localization of GLUT2 in β-cells and found that the co-localization of GLUT2-GFP with lysosome is significantly increased in the FA2H KO cells (Fig. 3, *I*–*K*). Treatment of cells with the lysosomal inhibitor bafilomycin effectively prevented the reduction of GLUT2 levels caused by FA2H KO (Fig 3*L*), supporting that FA2H deficiency promotes GLUT2 transport to the lysosomes for degradation and results in decreased GSIS.

### FA2H overexpression stabilizes GLUT2 protein

**Figure 4:**
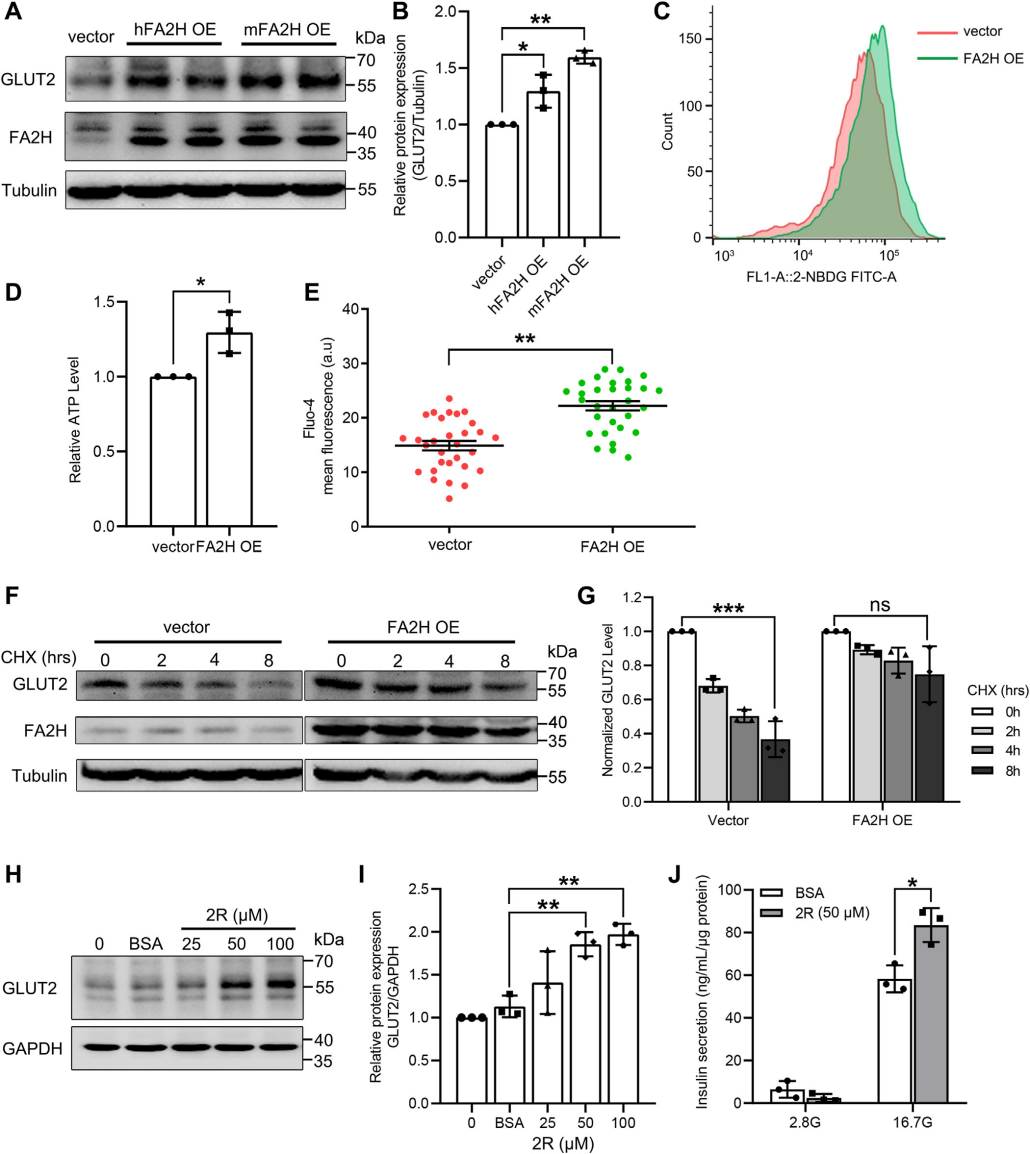
**FA2H overexpression stabilizes GLUT2 protein.***A* and *B*, whole-cell lysates of MIN6 cells transfected with empty vector (vector) and cells stably overexpressing human FA2H (hFA2H OE) or mouse FA2H (mFA2H OE) were analyzed by Western blotting using antibodies as indicated. *C*, vector and FA2H OE cells were incubated with 30 μmol/L fluorescent glucose analog 2-NBDG, and their uptake was assayed by flow cytometry. *D*, intracellular ATP levels in vector and FA2H OE cells (n = 3 biological experiments). *E*, intracellular Ca^2+^ levels were measured with the Ca^2+^-dependent fluorescent dye Fluo-4 (n = 33 cell clusters). *F* and *G*, vector and FA2H OE cells were treated with 100 mg/ml CHX for 2, 4, and 8 h, and the whole-cell lysates were examined by Western blotting using antibodies as indicated. The band intensities of three independent experiments were quantified by ImageJ. *H* and *I*, MIN6 cells were treated with different concentrations of (*R*)-2-OHPA (2R) for 24 h. Cell lysates were prepared and subjected to Western blot analysis with antibodies as indicated. The band intensities of three independent experiments were quantified by ImageJ. *J*, GSIS (at low [2.8 mM, 2.8 G] and high [16.7 mM, 16.7 G] glucose conditions) in MIN6 cells pretreated with 50 μM (*R*)-2-OHPA (2R) or BSA control (n = 3 biological experiments). Values represent mean ± SD. ∗, *p* < 0.05, ∗∗, *p* < 0.01, ∗∗∗, *p* < 0.001, ns, not significant by Student’s *t* test (*D*, *E*), one-way (*B*, *I*) or two-way (*G*, *J*) ANOVA.

To confirm the FA2H regulation of GLUT2, we examined its protein levels in MIN6 cells over-expressing human or mouse FA2H. Our results showed that total GLUT2 protein levels are significantly increased in both lines of FA2H OE cells as compared to control vector cells (Fig. 4, *A* and *B*) with increased uptake of 2-NBDG (Fig. 4*C*). Moreover, the ATP levels and Ca^2+^ levels are also increased dramatically in FA2H OE cells (Fig. 4, *D* and *E*). Consistent with enhanced lysosomal GLUT2 degradation in the absence of FA2H, GLUT2 protein exhibits slower degradation kinetics in FA2H OE cells in the presence of cycloheximide (Fig. 4, *F* and *G*), suggesting that FA2H stabilizes GLUT2 protein and represents a promising target to improve β-cell functions. We further investigated whether the enzymatic products of FA2H, (*R*)-2-OH FAs, have similar effects to FA2H and found that treatment with (*R*)-2-OH PA in MIN6 cells significantly increases the protein level of GLUT2 (Fig. 4, *H* and *I*) and enhances GSIS at 50 μM (Fig. 4*J*).

### FA2H regulation of insulin release and glucose tolerance is hydroxylation-dependent

The residues TYR322 and ASP323 of Scs7p, a yeast homolog of FA2H, are critical determinants of the hydroxylase reaction (Fig. S5*A*) (31). To confirm whether the observed FA2H regulation of GSIS is hydroxylation dependent, we generated a mouse FA2H mutant with the corresponding residues TYR311 and ASP312 replaced by alanine (FA2H MUT). We then constructed recombinant adeno-associated virus serotype 8 (rAAV8) encoding FLAG-tagged wild-type FA2H (FA2H WT) or FA2H MUT under the mouse insulin promoter (MIP). The rAAV8-MIP-FA2H WT and rAAV8-MIP-FA2H MUT were specifically overexpressed in MIN6 cells, but not in 293T cells (Fig. S5*B*), which was further confirmed by immunofluorescence analysis (Fig. S5*C*). Over-expression of FA2H WT significantly increases levels of 2-OHFAs, while FA2H MUT has minimal effects (Fig. S5*D*). Moreover, the overexpression of FA2H WT efficiently catalyzed the 2-hydroxylation of D4-PA to generate D4-2-OHPA, while FA2H MUT has minimal activity (Fig. S6). In contrast to FA2H WT (Fig 1*D*), over-expression of FA2H MUT does not influence GSIS in MIN6 cells (Fig. S7).

**Figure 5:**
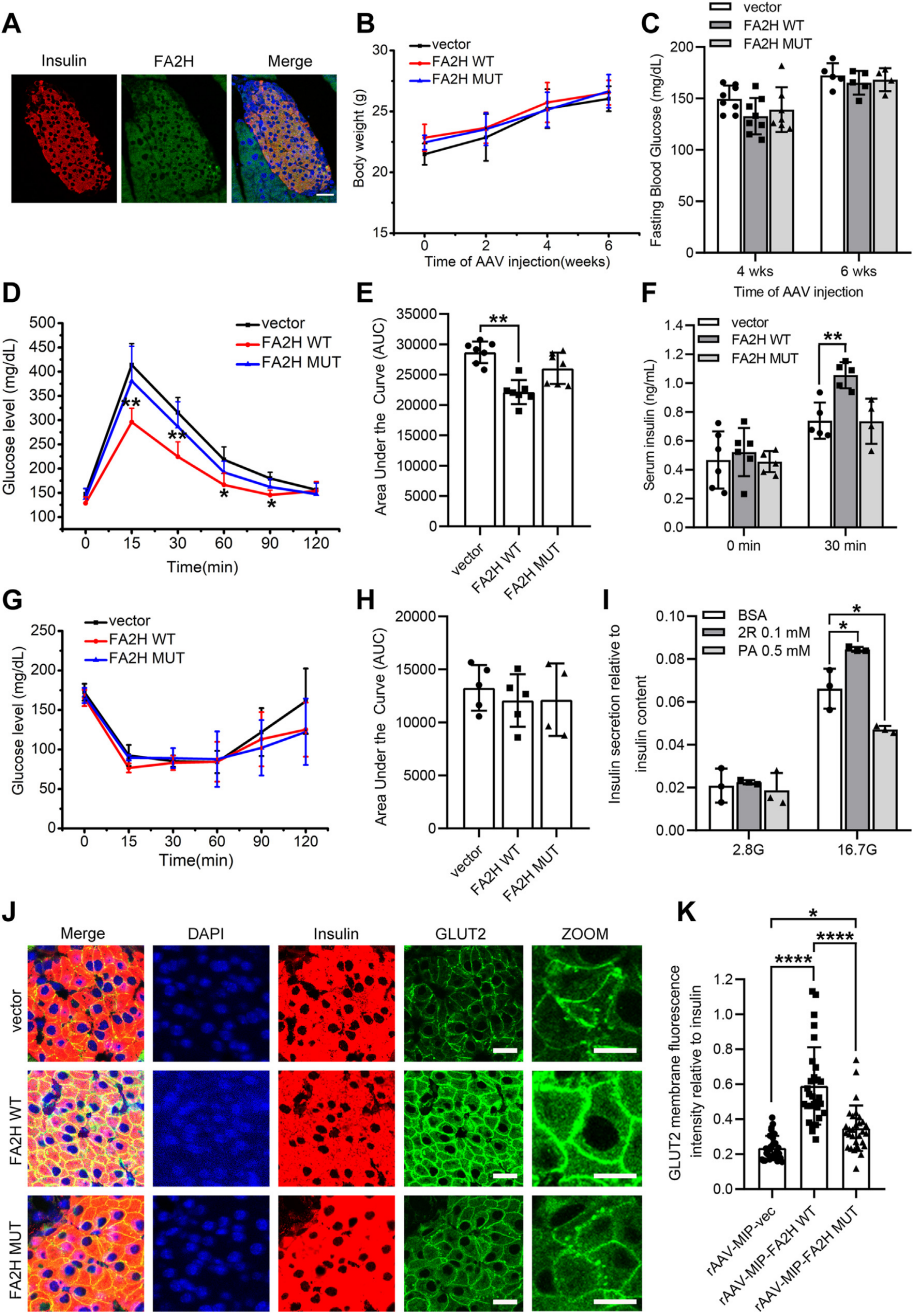
**FA2H increases glucose tolerance and GSIS in mice.***A*, immunofluorescence staining of Insulin (*red*) and FA2H (*green*) in pancreatic tissue. Scale bar, 25 μm. *B* and *C*, body weight and fasting blood glucose levels of mice infected with rAAV8-MIP-vector, rAAV8-MIP-FA2H WT, or rAAV8-MIP FA2H MUT (n = 8 mice per group). *D* and *E*, blood glucose levels (*D*) and AUC (*E*) of glucose tolerance test in mice infected with rAAV8-MIP-vector, rAAV8-MIP-FA2H WT, or rAAV8-MIP-FA2H MUT (2 g glucose/kg body weight, n = 8 mice per group). *F*, serum insulin levels of overnight fasted mice injected with glucose solution (2 g glucose/kg body weight, (n = 7 mice per group)). *G* and *H*, bected with rAAV8-MIP-vector, rAAV8-MIP-FA2H WT, or rAAV8-MIP-FA2H MUT (1 IU insulin/kg body weight, n = 5 mice per group). *I*, GSIS (at low [2.8 mM, 2.8 G] and high [16.7 mM, 16.7 G] glucose conditions) of islets isolated from WT mice preincubated with (*R*)-2- OHPA (2R) or PA (n = 3 biological experiments). *J* and *K*, GLUT2 signal from different groups was examined by a fluorescence microscope. The membrane and total cell fluorescent density of GLUT2 were quantified by ImageJ (n = 30 cell clusters). Scale bar, 5 μm. Values represent mean ± SD. ∗, *p* < 0.05, ∗∗, *p* < 0.01, ∗∗∗∗, *p* < 0.0001 by one-way (*B*, *D*, *E*, *G*, *H*, *K*) or two-way (*C*, *F*, *I*) ANOVA.

The IHC staining of mouse pancreatic tissue showed that FA2H protein is expressed in pancreatic islets (Fig. S8*A*). *In situ* immunofluorescence double staining of pancreatic tissue sections showed that FA2H was co-expressed with insulin in mouse pancreatic β-cells (Fig. 5*A*). To gain insights into the role of FA2H in the pancreas, we injected mice with rAAV8-MIP-FA2H WT or rAAV8-MIP MUT to induce β cell-specific overexpression of FA2H proteins. Western blot analysis confirmed the specific overexpression of the FA2H proteins in the pancreas (Fig. S8*B*). Overexpression of FA2H WT or MUT does not affect body weight or fasting glucose level (Fig. 5, *B* and *C*). No differences in the β cell mass and distribution of α and β cells in islets were observed (Fig. S8, *C*–*E*). Intraperitoneal glucose tolerance significantly improves in mice over-expressing FA2H WT in β cells but not in mice over-expressing FA2H MUT (Fig. 5, *D* and *E*). This may be explained by the enhanced glucose-induced increase of blood insulin level in mice over-expressing FA2H WT. Consistent with glucose tolerance results, over-expression of FA2H MUT has no effects on blood insulin levels (Fig. 5*F*). To examine whether the improved glucose tolerance in mice over-expressing FA2H WT is associated with insulin sensitivity in metabolic tissues, we performed an insulin tolerance test and found no difference in mice over-expressing FA2H WT or MUT (Fig. 5, *G* and *H*). We next examine the potential effects of FA 2-hydroxylation on isolated mouse islets and found that preincubation with (*R*)-2-OH PA increases GSIS while PA decreases it as anticipated (Fig. 5*I*). These data suggest that pancreatic β cell-specific FA2H-derived hydroxylation enhances GSIS and improves pancreatic functions. Moreover, GLUT2 on the plasma membrane is significantly increased by overexpression of FA2H WT. In contrast, the effect of MUT overexpression is much weaker (Fig. 5, *J* and *K*), suggesting that regulation of GLUT2 may contribute to the increased GSIS *in vivo* by FA2H overexpression.

### Regulation of FA2H in the pancreas in T2D

**Figure 6:**
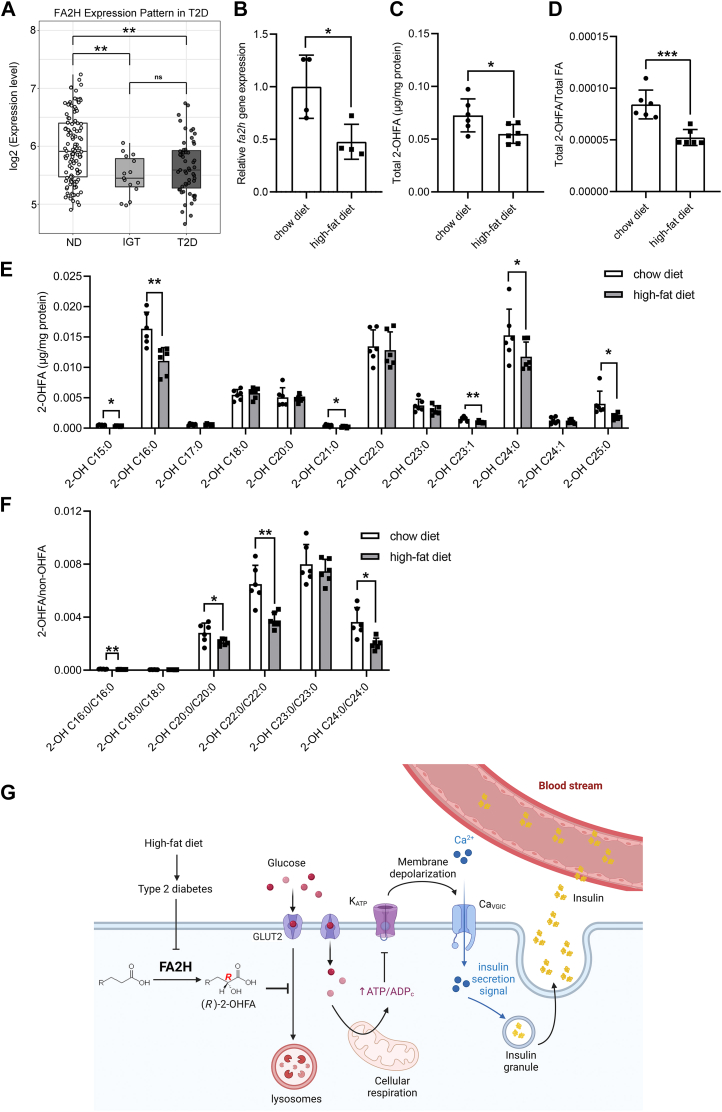
**Regulation of FA2H in the pancreas in T2D.***A*, FA2H gene expression in T2D, IGT (impaired glucose tolerance), and non-diabetic (ND) islets in published microarray dataset GSE76896. *B*, qRT-PCR analysis of the FA2H gene in the pancreas of mice fed either a chow or high-fat diet (n = 4 mice per group). *C*, GC-MS analysis of total 2-OHFAs in the pancreas of mice fed either a chow or high-fat diet (n = 6 mice per group). *D*, the ratio of total 2-OHFAs to total FAs in the pancreas of mice fed either a chow or high-fat diet. *E*, GC-MS analysis of individual 2-OHFA species in the pancreas of mice fed either a chow or high-fat diet (n = 6 mice per group). *F*, the ratios of individual 2-OHFA species to their corresponding non-hydroxy FAs in the pancreas of mice fed either a chow or high-fat diet. *G*, a proposed model for FA2H regulation of GSIS. FA2H catalyzes the conversion of fatty acids to chiral-specific (*R*)-2-OHFAs. Levels of *Fa2h* gene expression and 2-OHFAs are lower in HFD-induced obesity and T2D. FA2H-mediated generation of (*R*)-2-OHFAs is important to maintain GLUT2 on the plasma membrane by blocking its internalization and trafficking to the lysosomes for degradation, leading to enhanced GSIS. Created with BioRender.com. Values represent mean ± SD. ∗, *p* < 0.05, ∗∗, *p* < 0.01, ∗∗∗, *p* < 0.001, ∗∗∗∗, *p* < 0.0001 by Student’s *t* test (*B*–*F*) or one-way (*A*) ANOVA.

We obtained a set of public transcriptome data (GSE76896) of pancreatic islet tissues from the Gene Expression Omnibus database with available FA2H expression levels (32, 33). Interestingly, the expression of FA2H is significantly lower in impaired glucose tolerant (IGT) and T2D islets when the insulin secretion index is significantly suppressed than in non-diabetic islets (Fig. 6*A*), indicating potential involvement of FA2H in human β cell functions. We next examined the regulation of the *Fa2h* gene in mouse pancreas with T2D induced by an HFD for 6 months (34). HFD decreases the *Fa2h* expression level (Fig. 6*B*) and 2-OHFAs (Fig. 6, *C* and *D*), with the most significant decreases observed in 2-OH C16:0, 2-OH C24:0 and 2-OH C25:0 (Fig. 6*E*). Interestingly, the ratios of 2-OHFAs to their corresponding non-hydroxy FAs are much higher in very long-chain FAs (*e.g*. C24:0) than in long-chain FAs (*e.g*. C16:0) (Fig. 6*F*), indicating that very long-chain FAs may be the preferred substrates for FA2H in the pancreas. Collectively, our results suggest that FA2H may be involved in the β cell dysfunction during the progression of T2D (Fig. 6*G*).

## Discussion

*FA2H* mutations cause SPG35/FAHN, which has been associated with various neuronal diseases characterized by intellectual decline and problems with movement, vision, and speech (13). Its potential functions in keratinocyte differentiation, spermatogenesis, adipogenesis, and animal development have been demonstrated (13, 35). *FA2H* expression was detected in the pancreas with unknown functions when initially cloned and characterized (36). Our current study revealed a function of FA2H in pancreatic insulin secretion and hormonal regulation of whole-body glucose homeostasis (Fig. 6*G*). Most previous studies emphasize the importance of 2-OHFAs in the FA2H functions. Our data demonstrate that FA2H-mediated hydroxylation is directly linked to insulin secretion by preventing the internalization and trafficking of GLUT2 to lysosomes for degradation.

There are two types of FA 2-hydroxylases in mammalian cells to date: the 2-oxoglutarate-dependent oxygenase phytanoyl-CoA 2-hydroxylase and the di-iron-containing monooxygenase FA2H (13). FA2H is generally considered the only 2-hydroxylase known to utilize straight long-chain FAs as substrates in humans. Surprisingly, skin fibroblasts of the patients with a deleterious mutation at the *FA2H* gene have slightly higher FA 2-hydroxylation activity than control cells (37). The amounts of sphingomyelin species containing 2-OHFAs are normal in patient lymphocytes (38), indicating the presence of additional enzymes with the FA 2-hydroxylation activity in human cells, and their identification and characterization would help uncover additional FA metabolic pathways regulating GSIS. The pathways responsible for the degradation of 2-OHFAs have not been fully understood, which may involve the peroxisomal 2-hydroxyacyl-CoA lyase 1 (HACL1), α-hydroxy acid oxidase 1 and 2 (HAOX1 and HAOX2) (39), and HACL2 in the ER (40). In the pancreas, HAOX1 is the predominant form of HAOX (41), while both HACL2 and HACL1 are detected (40). A potential GSIS regulation by HACL1/2 and HAOX1 is anticipated but likely depends on their expression levels and substrate specificities, which warrants future investigation.

Closure of K_ATP_ requires both ATP and H_2_O_2_, indicating that metabolic and redox signaling integration is fundamental for GSIS (42). NADPH facilitates GSIS *via* the production of cytosolic H_2_O_2_ by NADPH oxidase isoform 4 (NOX4) (43) and glutaredoxin-mediated activation of sentrin/SUMO-specific protease-1 (SENP1), a de-SUMOylase enzyme that targets insulin granule exocytosis secretagogue-driven secretory proteins (44). Reductive TCA cycle flux *via* IDH2 produces cytosolic NADPH as an additional metabolic output that stimulates insulin granule exocytosis. FA2H contains an N-terminal cytochrome b5 domain, and its activity is NADPH sensitive (36), suggesting that part of NADPH-mediated GSIS may be due to regulation of FA 2-hydroxylation. Moreover, hypoxia suppresses the expression of FA2H, altering the structure of the plasma membrane and lipid rafts (45). The increased anaerobic glycolytic flux, independent of glucose levels, impairs GSIS during postprandial hyperglycemia (46), which could be due to the suppressed FA2H expression by hypoxia. Moreover, the degradation of 2-OHFA also generates compartmentalized H_2_O_2_, suggesting that 2-OHFA metabolism may integrate nutrient sensing and redox homeostasis in GSIS.

GLUT2 is the predominant glucose transporter expressed in rodent pancreatic β cells (29). The GLUT2 trafficking and degradation are regulated by *N*-linked glycosylation (20) and tubule fission (21). Lipid regulation of GLUT2 trafficking was revealed by a study using 1-palmitoyl-2-linoleoyl-3-acetyl-*rac*-glycerol (PLAG) originally isolated from the antlers of Sika deer. PLAG accelerates GLUT2 internalization and reduces the entry of glucose and streptozotocin by an unknown mechanism (47). Our current study demonstrated that FA2H knockdown promotes endocytosis and trafficking of GLUT2 to lysosomes for degradation *via* the caveolae-dependent pathway, providing a new perspective correlating structural modification of FA to glucose sensing in pancreatic β cells. Our previous studies revealed that FA2H regulates GLUT4 trafficking and degradation with minimal effect on GLUT1 in adipocytes (12, 16). Moreover, FA2H partially inhibits tumor growth by impacting GLUT1 level and glucose sensing by AMPK (14, 15). High glucose induces rapid GLUT2 internalization and degradation in β-cells while GLUT2 expressed in 3T3-L1 adipocytes remains on the cell surface and is resistant to degradation (30). These results indicate that FA2H regulates various glucose transporters presumably through cell-type-specific mechanisms, which warrant future investigation.

Early diabetes and abnormal postnatal pancreatic islet development are observed in GLUT2-deficient mice (48), which can be restored *via* the pancreatic-specific expression of GLUT2 (18). Moreover, numerous obese and lean diabetes models exhibit reduced GLUT2 expression in rodents, suggesting that the suppressed GLUT2 plays an essential role in the development of T2D (29). Different transporters' contribution to glucose utilization is more complicated and controversial in human β-cells. Many studies argue that GLUT1 and GLUT3 are the predominant glucose transporters, supported by human islet cells' low glucose uptake velocity due to a low K_m_ transporter such as GLUT1 or GLUT3 (49). However, GLUT1 and GLUT3 reach their maximum glucose transport velocity already at basal glucose levels in humans (3.9–6.1 mM), and the adaption of glucose uptake to changes at high glucose levels (5–20 mM) may require the participation of low-affinity transporters like GLUT2. Indeed, the knockdown of either *GLUT1* or *GLUT2* shows no marked alterations in glucose transport and GSIS in human islet cell cultures, while their simultaneous knockdown significantly leads to the reductions (50). The discrepancy may be partially explained by the heterogeneous expression and function of GLUT2 in human pancreatic islets (51). Nevertheless, a recent study in maturity-onset diabetes of young 3 (MODY3) patients revealed that the decreased GLUT2 and glucose uptake contribute to insulin secretion defects (52).

Our study demonstrates that FA2H-mediated hydroxylation is causally linked to insulin secretion, highlighting potential clinical implications of FA2H dysregulation in T2D. However, the regulation of the FA2H gene and its protein stability in pancreatic β cells remains poorly understood. The role of cofactors, such as cytochrome P450 reductase and NADPH, in modulating FA2H hydroxylation activity within cells also remains unclear. Gaining insights into the molecular mechanisms that regulate FA2H levels and activity could pave the way for novel therapeutic strategies targeting FA2H in T2D. Pancreatic β cell 2-OHFAs can be synthesized endogenously by FA2H or absorbed from the plasma. The presence of 2-OHFAs in human plasma (53) suggests that peripheral 2-OHFAs might also contribute to the regulation of GSIS *in vivo*. Previous analyses have shown that seafood, particularly sea cucumbers, contain high levels of 2-OHFAs (54). Interestingly, higher total fish intake has been associated with a significantly lower risk of diabetes (55), and sea cucumber has been reported to have anti-diabetic and glucose-lowering properties (56). These findings support the idea that extracellular sources of 2-OHFAs might contribute to the regulation of GSIS in human islets and dietary supplementation with 2-OHFAs may represent a promising therapeutic approach. Moreover, analysis of 2-OHFAs in metabolically unhealthy obese (MUO) and metabolically healthy obese (MHO) subjects who have a normal HOMA-IR and no components of metabolic syndrome (57) will provide valuable human clinical data to validate our findings.

The minimal concentrations of (*R*)-2-OHFAs required to stabilize GLUT2 in MIN6 cells and enhance GSIS in islets (50 μM in this study) are approximately one order of magnitude higher than their physiological concentrations in human plasma (53). Plasma also contains significant amounts of 2-OHFAs incorporated into lipid species *via* ester or amide bonds, which could lead to high localized concentrations of free 2-OHFAs through enzymatic hydrolysis. Nevertheless, these levels are consistent with those used in studies employing natural lipids as pharmacological agents, often 1 to 2 orders of magnitude higher than their physiological levels (58). The need for such elevated concentrations may reflect challenges in delivering lipophilic compounds, which can aggregate or bind nonspecifically to other proteins.

There are some limitations to this study. First, the specific mechanisms responsible for FA2H regulation of GLUT2 endocytosis and trafficking to lysosomes are unclear. Since 2-OHFAs can either exist as FFAs or be incorporated into more complex lipids, this regulation may involve the generation of particular lipids species containing (*R*)-2-OHFA interacting with endocytic machinery. Identifying the lipid species responsible for the observed FA2H functions requires the synthesis of standards and subsequent verification of their effects. Second, glucokinase is a metabolic sensor involved in the regulated release of insulin, and its activator, GKA50, enhances GSIS in a glucose-dependent manner with increased EC50 values without affecting the maximal rates of GSIS (19). The molecular coordination of glucose uptake and phosphorylation in GSIS under different pathophysiological conditions represents an important area for future study. Third, to our knowledge, no GWAS studies have directly linked FA2H to glucose metabolism, likely due to the low frequency of relevant variants in the general population, which makes it difficult for GWAS to detect significant correlations. SPG35/FAHN is a rare subtype of hereditary spastic paraplegia. A comprehensive metabolic evaluation, including glucose metabolism in affected individuals, has not yet been reported and warrants further investigation to better understand the metabolic implications of the disease. Finally, the decreased FA2H expression levels in IGT and T2D subjects whose insulin secretion is impaired suggests that FA2H may be involved in the regulation of glucose transporters and human β cell physiology, but this conclusion needs to be confirmed in human pancreatic β cell lines and islets isolated from human subjects. Further clinical correlation between FA2H expression and β cell function would significantly strengthen this association. Nevertheless, our data revealed that FA2H-mediated hydroxylation is causally related to insulin secretion. The identified chemical switch linking FA metabolism to GSIS may help better understand the mechanisms by which β cells integrate different nutrients to regulate insulin secretion and shed new insights into the pathogenesis of metabolic diseases.

## Experimental procedures

### Cell culture

MIN6 cells were provided by Dr S. Seino (Kobe University, Japan) and cultured in high glucose DMEM (Hyclone) supplemented with 15% FBS (Gibco), 2.5 μl/L β-mercaptoethanol (Sigma), 200 U/ml penicillin, 50 μg/ml streptomycin (Gibco) and 10 mM HEPES pH 7.2. HEK293T cells (ATCC) were cultured in high glucose DMEM supplemented with 10% FBS, 200 U/ml penicillin, and 50 μg/ml streptomycin.

### CRISPR/Cas9-mediated FA2H KO

FA2H-specific and control CRISPR Double Nickase Plasmids were purchased from Santa Cruz Biotechnology (sc-436124-NIC and sc-437284, respectively). The plasmid mixtures were transfected into MIN6 cells using Lipofectamine 2000 followed by puromycin selection according to the manufacturer’s protocols.

### Plasmids and viruses

Mouse *Glut2* gene was amplified by PCR from the cDNA of MIN6 cells and cloned into a pEGFP C1 vector. Mouse FA2H genes were amplified by PCR from pcDNA-3.1-FA2H plasmid and cloned into a pLenti vector. All constructs were confirmed by DNA sequencing. Lentivirus encoding mouse FA2H was generated by a triple plasmid lentiviral packaging technique as previously described (14). PCR-based site-directed mutagenesis was performed to introduce Y311A/D312A mutation in mouse FA2H using QuikChange Site-Directed Mutagenesis. The PCR products were cloned into pFBD AAV vector containing the MIP, and the rAAV8 viral particles were generated by Suzhou Genehealth Biotechnology. Primers used for PCR were listed in Table S1.

### RNA extraction and qRT-PCR

Total RNAs were extracted from MIN6 cells using TRIzol reagent (Invitrogen). The cDNA was synthesized using PrimerScript RT Master Mix (TaKaRa), and qRT-PCR was performed using SYBR Green RT-PCR kits (TaKaRa) as previously described (14). Primers were listed in Table S2.

### Protein extraction and Western blotting

Cells were lysed in ice-cold lysis buffer (50 mM Tris-HCl pH 7.5, 150 mM NaCl, 1 mM EDTA, 1% Triton X-100) containing protease inhibitor cocktail (Sigma) and the whole-cell lysates were clarified by centrifugation (4 °C, 12,000 rpm, 10 min). Proteins were separated by SDS-PAGE, transferred to nitrocellulose filter membrane, and immunoblotted as previously described (14). The proteins were visualized by chemiluminescence, and signals were quantified using ImageJ software. The specificity of the FA2H antibody was validated using a gene knockout model, ensuring the absence of signal in FA2H-deficient cells. Other antibodies used in the study were validated by the vendors, confirming their reliability through standard validation procedures, such as Western blotting or immunohistochemistry, as provided by the manufacturers. Antibody information was provided in Table S3.

### Immunohistochemistry and immunofluorescence staining

Immunohistochemistry and immunofluorescence staining was performed as previously described (14). Cells were incubated with MitoTracker or LysoTracker (Invitrogen) for 30 to 60 min following the manufacturer’s instructions.

### GSIS and KSIS

MIN6 cells were seeded at a 1 × 10^5^/well density in a 12-well dish. Cells were incubated in KRB containing 0.1% BSA and 2.8 mM glucose for 1 h, followed by stimulating with KRB plus 2.8 mM, 16.7 mM glucose or 20 mM KCl for 1 h. The supernatant was collected, and insulin levels were measured with an ELISA kit (Elabscience).

### Glucose uptake

Glucose uptake in MIN6 cells was measured as described (59) with 2-NBDG (Invitrogen, 30 μmol/L). The cells were run on a CytoFlex flow cytometer (Beckman) and analyzed using FlowJo-V10 software.

### Intracellular Ca^2+^ measurement

MIN6 cells were seeded into glass-bottomed chambers (NEST, 20 mm) at a 1 × 10^5^/well density and cultured in high glucose DMEM medium for 24 h. To measure intracellular Ca^2+^, Fluo4-AM (Beyotime Biotechnology) fluorescent dye solutions were added into the Ca^2+^-free KRBH buffer and stimulated by 16.7 mM glucose for 30 min. Images were analyzed using software under a live-cell imaging system (Leica) by measuring fluorescence at 488 nm.

### Cellular ATP assay

MIN6 cells were incubated with 16.7 mM glucose for 30 min. The relative ATP levels were measured using an enhanced ATP assay kit (Beyotime Biotechnology) following the manufacturer’s instructions.

### FA and 2-OHFA analysis

Total lipids were extracted using the Bligh-Dyer method, and FA methyl esters were prepared and analyzed as described (60). 2-OH FA methyl esters were enriched by solid-phase extraction and further derivatized to trimethylsilyl ethers before GC/MS analysis as described (54).

### Mice

C57BL/6J mice were purchased from Shanghai SLRC Laboratory Animal Co., Ltd and maintained under SPF conditions. Mice were housed 3 to 5 in a cage with unrestricted access to water, within a controlled environment set at a temperature of 25 ± 2 °C and a humidity level of 60 to 70%. AAV infections were carried out in male C57BL/6J mice (6–8 weeks old, 18–25 g) randomly divided into three groups at a dose of 1 × 10^12^ (rAAV8-MIP-vector, rAAV8-MIP-FA2H WT, or rAAV8-MIP-FA2H MUT) viral particles per mouse. Viruses were administered by i.p. injection in a total volume of 100 μl. Mice exclusion from the analysis was due to mortality during the experiment. For HFD feeding, 8-week-old male C57BL/6J mice (20–25 g) were randomly chosen and fed with either a chow diet (10 kcal% fat; D12450J; Research Diets) or an HFD (60 kcal% fat; D12492; Research Diets) for 6 months. All mouse procedures were approved by the Committee on the Ethics of Animal Experiments of Soochow University (Suzhou, China).

### Mouse islet isolation and insulin secretion assay

Mouse islets were isolated from the pancreas of 2% pentobarbital sodium (Sigma-Aldrich) sedated 8- to 12-week-old male C57BL/6J mice by 1 mg/ml collagenase type V (Sigma-Aldrich) digestion and purified by Histopaque 1077 (Sigma-Aldrich) density-gradient centrifugation as previously described (34). Before insulin secretion assay, isolated islets were cultured in RPMI 1640 medium (GIBCO) containing 5% FBS and 2 mM glucose for 24 h at 37 °C and then incubated in Krebs-Ringer Buffer (KRB) containing 0.1% BSA and 2.8 mM glucose for 30 min 10 islets were then transferred to a PCR tube containing 2.8 mM or 16.7 mM glucose (100 μl glucose solution and 10 islets) in KRB/0.1% BSA and incubated at a 37 °C water bath for 1 h. Supernatants were collected for insulin analysis using an ELISA kit (Ezassay) and normalized to insulin content in the islets extracted with acidified ethanol.

### Glucose tolerance, insulin sensitivity tests, and blood insulin level measurement

Glucose tolerance and insulin sensitivity tests were performed as described (21). Blood glucose levels were measured at indicated time points after intraperitoneal glucose injection (2 g/kg body weight) or human crystalline insulin (1 IU/kg, Eli Lilly). Blood insulin levels were taken by tail vein nick and measured using an ELISA kit (Elabscience) following the manufacturer’s protocol.

### Bioinformatics analysis

The transcriptome raw data of GSE76896 (32, 33) were downloaded from the Gene Expression Omnibus database (http://www.ncbi.nlm.nih.gov/geo/). The data were analyzed using the R (version 3.5.3) script and preprocessed by the "rma" function of the "oligo" package (version 1.44.0) in Bioconductor, including background correction and data normalization. The differential expression of *FA2H* between diabetic and non-diabetic islet samples was calculated by the "eBayes" function of the "LIMMA" package (version 3.6.5), and the Benjamini-Hochberg method was used to correct the *p* values.

### Statistical analysis

Data are expressed as mean ± SD and analyzed by repeated-measures ANOVA and unpaired Student’s *t* test with GraphPad Prism software (GraphPad Software, version 9). *p* < 0.05 denotes statistical significance. Normality and equal group variances were tested before using ANOVA and Student’s *t*-tests in all the analyses.

## Data availability

All data supporting the findings of this study are available within the main manuscript and supporting information file.

## Supporting information

This article contains supporting information.

## Conflict of interest

The authors declare that they have no conflicts of interest with the contents of this article.
