# Supplementary material for: 2-Hydroxylation is a chemical switch linking fatty acids to glucose-stimulated insulin secretion
Source: J Biol Chem. 2024 Oct 21;300(12):107912. doi: 10.1016/j.jbc.2024.107912 (PMC11742320; doi:10.1016/j.jbc.2024.107912)
Supplement: Supporting information [file mmc1.pdf]

2-Hydroxylation is a chemical switch linking fatty acids to glucose-stimulated insulin secretion

Supplement

Supplementary Figure S1. FA2H in MIN6 cells does not affect levels of insulin protein or mRNA.

Supplementary Figure S2. FA2H does not affect cytoskeleton remodeling.

Supplementary Figure S3. FA2H does not affect ER stress.

Supplementary Figure S4. FA2H deficiency increases caveolin-dependent internalization of GLUT2.

Supplementary Figure S5. Analysis of 2-OHFA species in MIN6 cells infected with rAAV8 encoding FA2H or its mutant.

Supplementary Figure S6. 2-hydroxylation activities of FA2H and its mutant.

Supplementary Figure S7. FA2H mutant does not affect GSIS in MIN6 cells.

Supplementary Figure S8. Mice infected with rAAV8 encoding FA2H and its mutant display a normal development of the pancreas.

Supplementary Table S1. Primers used for plasmid cloning by PCR

Supplementary Table S2. Primers used for qRT-PCR analysis.

Supplementary Table S3. Antibody information.

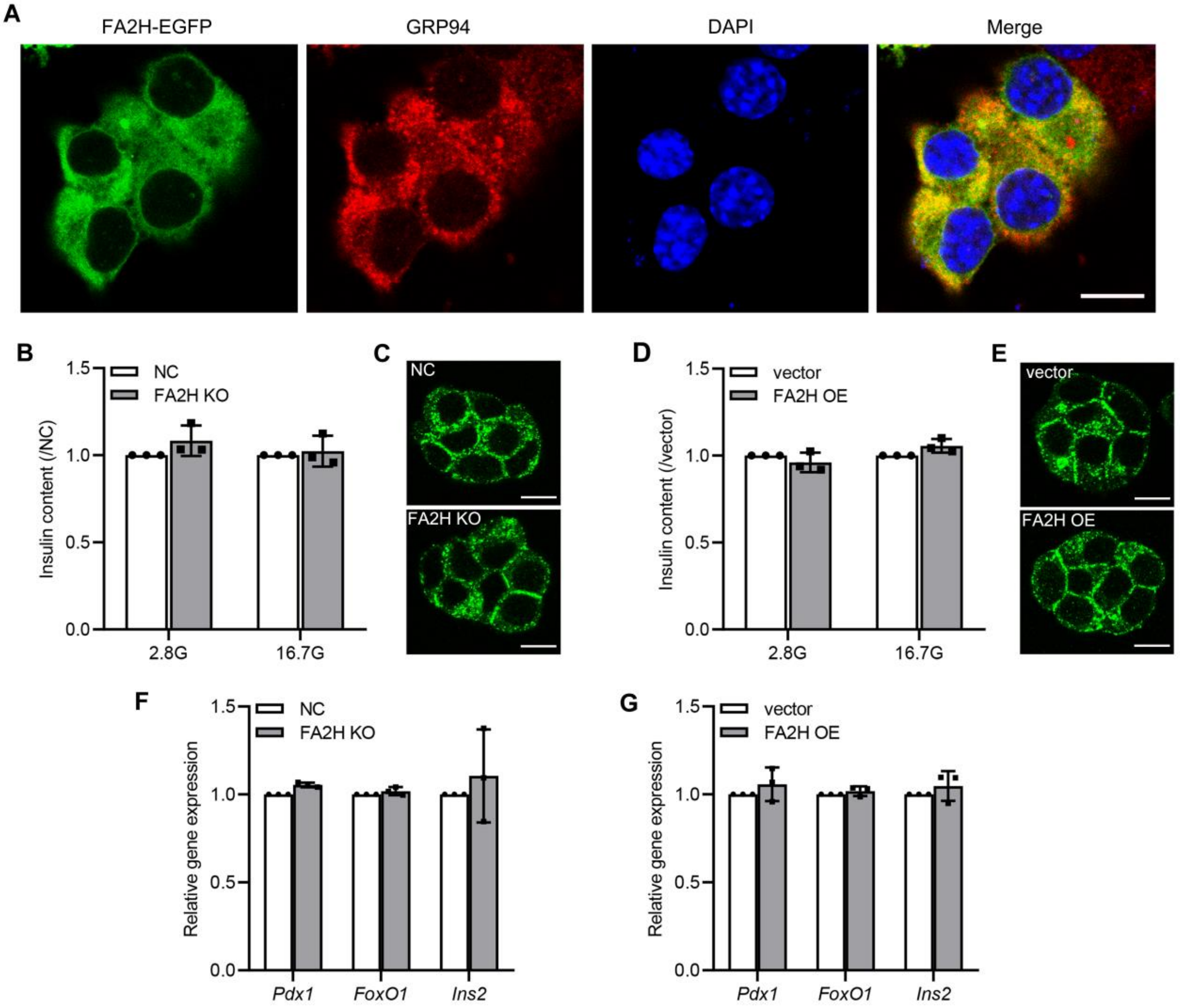

Supplementary Figure S1. FA2H in MIN6 cells does not affect levels of insulin protein or mRNA. A: Co-localization of FA2H (green) and ER marker GPR94 (red) in MIN6 cells. Scale bar, 10 µm. B: Total proteins of negative control (NC) cells and cells with FA2H knockout (KO) (at low [2.8 mM, 2.8G] and high [16.7 mM, 16.7G] glucose conditions) were extracted and insulin contents were measured by ELISA and normalized to total protein levels. C: Immunofluorescence staining of insulin in NC and FA2H KO cells. Scale bar, 10 µm. D: Total proteins of cells transfected with empty vector (vector) and cells stably over-expressing FA2H (OE) were extracted and insulin contents were measured by ELISA and normalized to total protein levels. E: Immunofluorescence staining of insulin in vector and FA2H OE cells. Scale bar, 10 µm. F, G: qRT-PCR analysis of the insulin synthesis-related genes in MIN6 cells. Values represent mean ± SD, n=3 biological experiments.

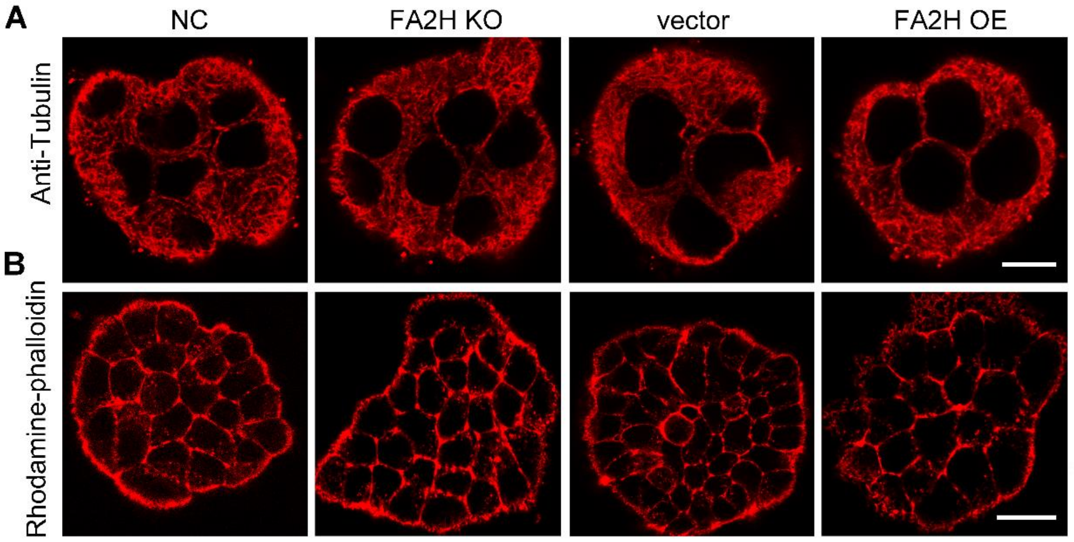

Supplementary Figure S2. FA2H does not affect cytoskeleton remodeling. A: Tubulin in the negative control (NC) MIN6 cells, cells with FA2H knockout (KO), transfected with empty vector (vector) and stably over-expressing FA2H (OE) was visualized with Alexa Fluor 594 conjugated anti-tubulin antibody. Scale bar, 10 µm. B: F-actin was stained with Alexa 568-phalloidin rhodamine. Scale bar, 10 µm.

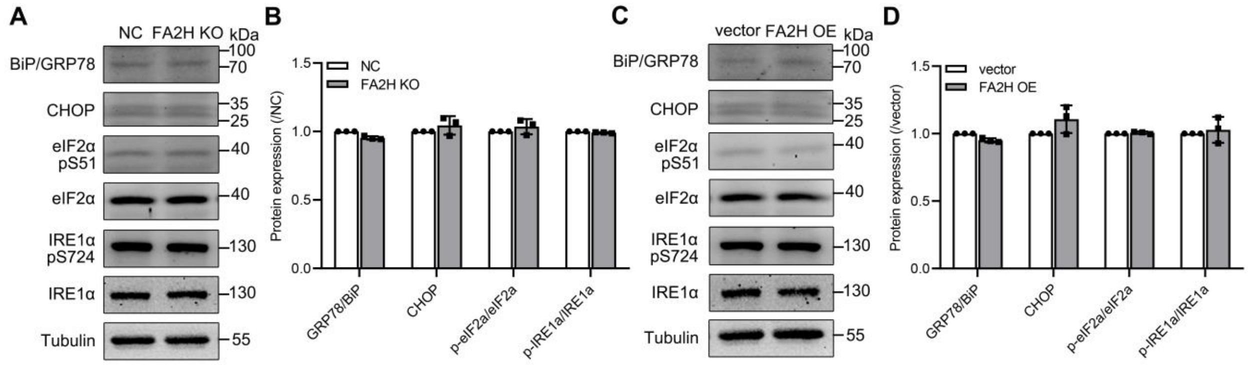

Supplementary Figure S3. FA2H does not affect ER stress. A: Whole-cell lysates of FA2H knockout (KO) or negative control (NC) MIN6 cells were analyzed by Western blotting using antibodies as indicated. B: The band intensities of three independent experiments in A were quantified by ImageJ. C: Whole-cell lysates of MIN6 cells stably over-expressing FA2H (FA2H OE) or transfected with empty vector (vector) were analyzed by Western blotting using antibodies as indicated. D: The band intensities of three independent experiments in C were quantified by ImageJ. Values represent mean ± SD, n=3 biological experiments.

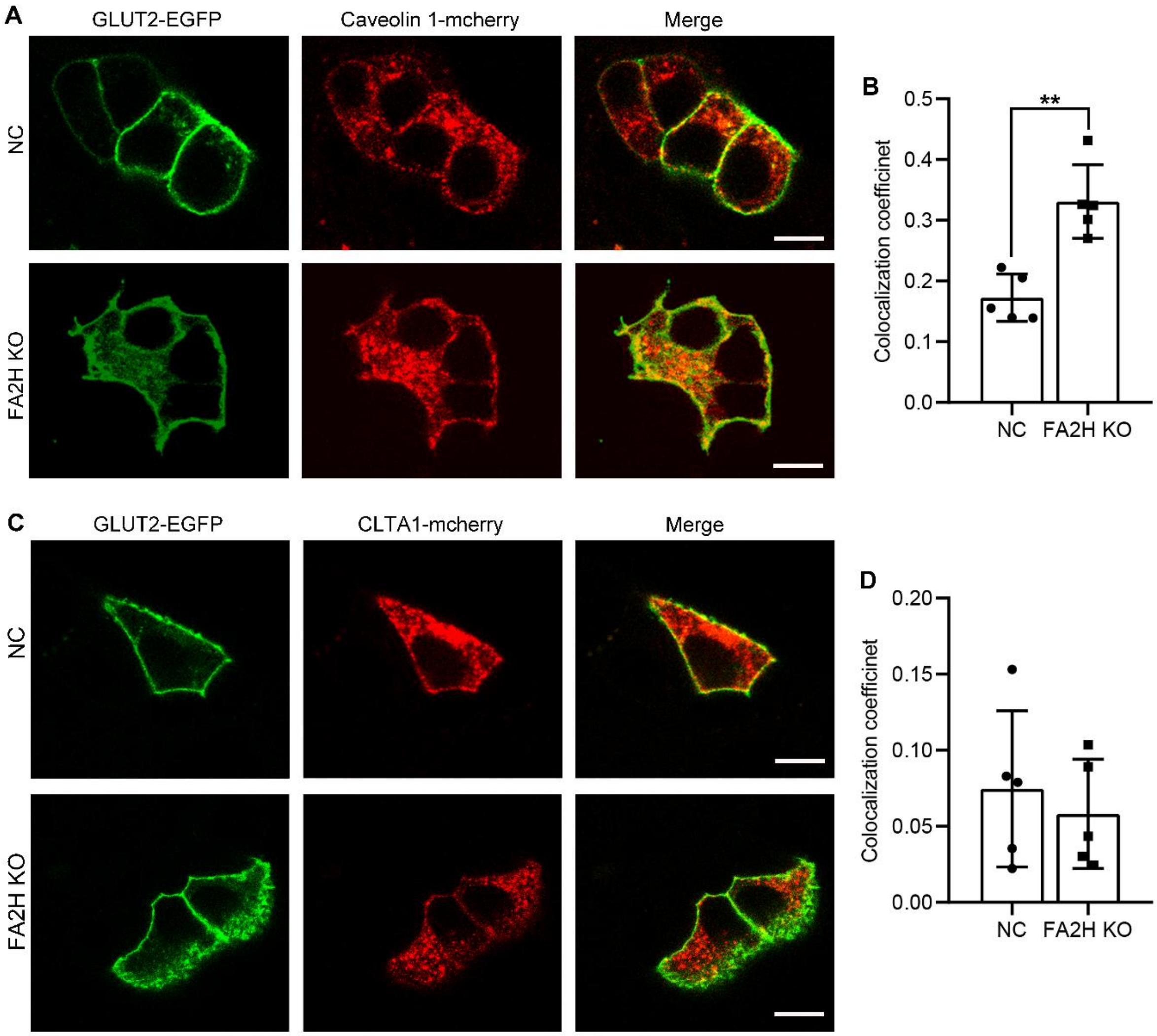

Supplementary Figure S4. FA2H deficiency increases caveolin-dependent internalization of GLUT2. A: Colocalization of GLUT2 (green) and caveolin1 (red) in NC and FA2H KO cells. Scale bar, 10 µm. B: Pearson correlation coefficients of GFP with caveolin in NC and FA2H KO cells (n=5 cell clusters). C: Colocalization of GLUT2 (green) and clathrin light chain (CLTA, red) in NC and FA2H KO MIN6 cells. Scale bar, 10 µm. D: Pearson correlation coefficients of (C) were presented as the mean ± SD (n=5 cell clusters). **, $p < 0.01$ by Student's *t*-test.

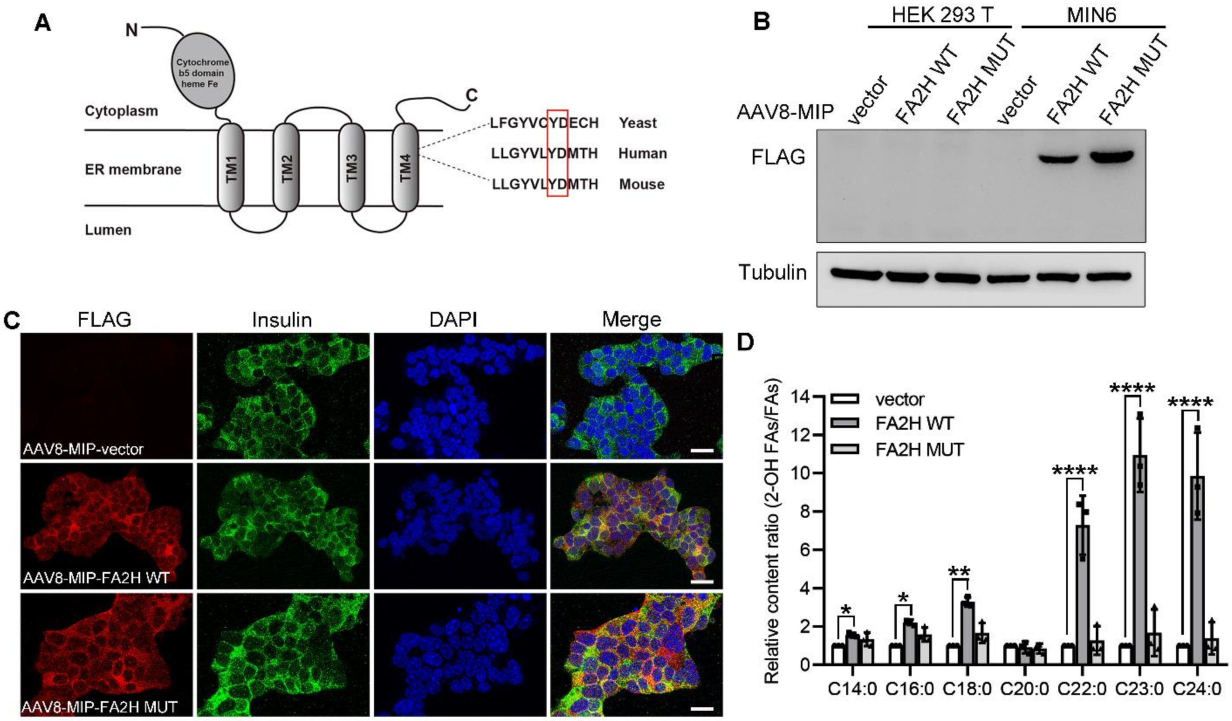

Supplementary Figure S5. Analysis of 2-OHFA species in MIN6 cells infected with rAAV8 encoding FA2H or its mutant. A: Sequence alignment of FA2H orthologs. B: HEK293 T and MIN6 cells infected with rAAV8 encoding wild-type (WT) FA2H, its mutant (MUT) with FLAG tag or empty vector (vector) under MIP were analyzed by Western blotting with antibodies as indicated. C: Immunofluorescence staining of FLAG and insulin in Min6 cells infected with indicated rAAV8. Scale bar, 20 μm. D: GC-MS analysis of 2-OHFAs in MIN6 cells infected with indicated rAAV8 (n=3 biological experiments). Values represent mean ± SD. *, $p < 0.05$, **, $p < 0.01$, ****, $p < 0.0001$ by one-way ANOVA.

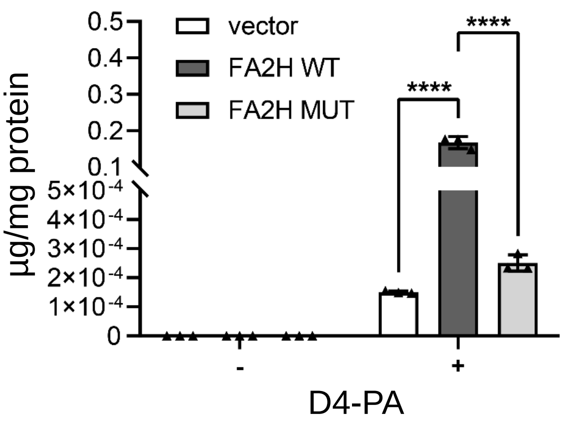

Supplementary Figure S6. 2-hydroxylation activities of FA2H and its mutant. CHO cells stably over-expressing wild type FA2H (FA2H WT), FA2H mutant (FA2H MUT) or empty vector (vector) were incubated with or without 100 µM D4-palmitic acid (D4-PA) for 12 hours. The generated D4-2-OHPA was derivatized and analyzed by GC-MS. Values represent mean ± SD, n=3 biological experiments, ****, $p < 0.0001$ by two-way ANOVA.

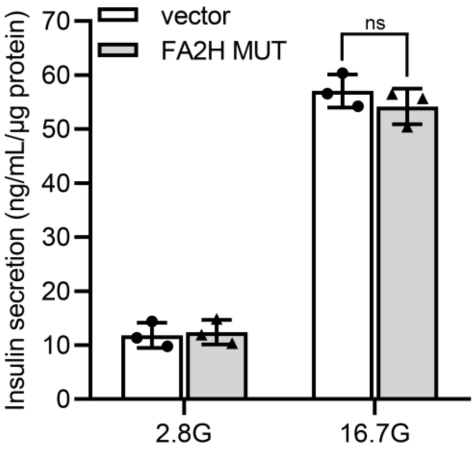

Supplementary Figure S7. FA2H mutant does not affect GSIS in MIN6 cells. GSIS (at low [2.8 mM, 2.8G] and high [16.7 mM, 16.7G] glucose conditions) in MIN6 cells stably over-expressing FA2H mutant (FA2H MUT) or empty vector (vector), n=3 biological experiments, ns, not significant by two-way ANOVA.

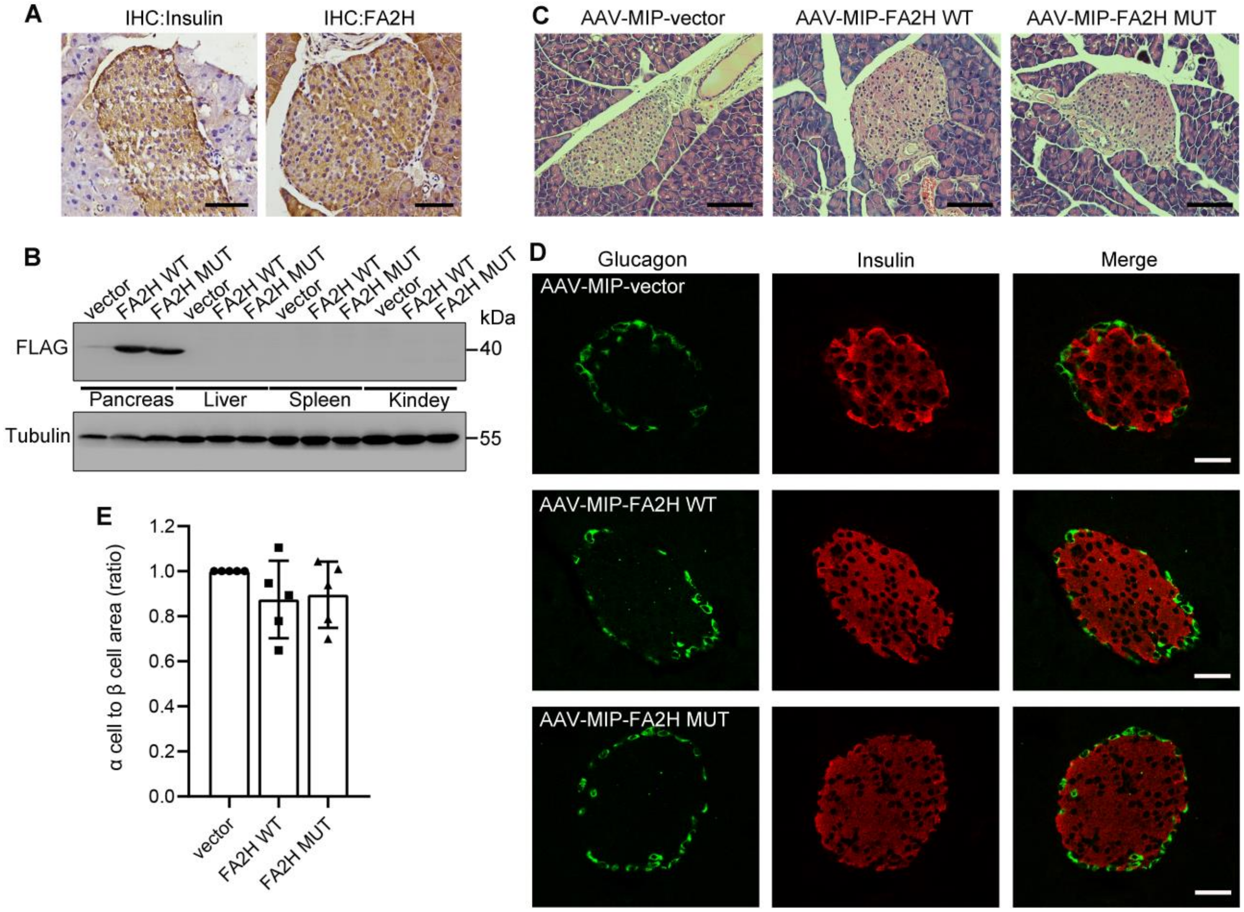

Supplementary Figure S8. Mice infected with rAAV8 encoding FA2H and its mutant display a normal development of the pancreas. A: Immunohistochemistry (IHC) staining of Insulin and FA2H in mouse pancreas tissue. Scale bar, 50 μm. B: The tissue protein samples of the mice infected with rAAV8-MIP-vector, rAAV8-MIP-FA2H WT, or rAAV8-MIP-FA2H MUT were analyzed by Western blotting with the indicated antibodies. C: Pancreatic sections of the indicated mice were subjected to H&E staining. Scale bar, 50 μm. D: Pancreatic sections of the indicated mice were subjected to immunofluorescence staining of glucagon (green) and insulin (red). Scale bar, 25 μm. E: The relative ratio of α cell to β cell was presented as the mean ± SD. n=5 mice per group by one-way ANOVA.

Supplementary Table S1. Primers used for plasmid cloning by PCR

Gene	Primer (5'-3')
m*Glut2*	Forward: GAGCATGTCAGATCTATGTCAGAAGACAAGATC
	Reverse: GTGACGTCAGGATCCTCATCCTCACACACTCTC
m*Fa2h*	Forward: GAGCATGTCCTCGAGATGGCCCCCGCTCCGCCC
	Reverse: GAGCATGTCGAATTCCTGCATCTTCGGGTGGGC
m*Fa2h* mutant	Forward: GCTGGGGGTCTCCTGGGCTATGTCCTCGCTGCCATGACACATTAC
	Reverse: AGAGCCGAAGTGCAGGTAGTAATGTGTCATGGCAGCGAGGACATA

Supplementary Table S2. Primers used for qRT-PCR analysis

Gene	Primer (5’-3’)
m*Pdx1*	Forward: AAATCCACCAAAGCTCACGC
	Reverse: CGAGGTCACCGCACAATCT
m*FoxO1*	Forward: GGGTCCCACAGCAACGATG
	Reverse: CACCAGGGAATGCACGTCC
m*Ins2*	Forward: CTGCTGGCCCTGCTCTTC
	Reverse: AACCACAAAGGTGCTGCTTGA
m*Glut2*	Forward: CTTGGTTCATGGTTGCTGAAT
	Reverse: GCAATGTACTGGAAGCAGAGG
m*Fa2h*	Forward: TCCCTAGTGATTGCCTTCTTCT
	Reverse: CATAGAGGACATAGCCCAGGAG
m*18S*	Forward: AGTTCCAGCACATTTTGCGAG
	Reverse: TCATCCTCCGTGAGTTCTCCA

Supplementary Table S3. Antibody information

Name	Company	Catalog Number	RRID	Dilution
FA2H	Proteintech	15452-1-AP	AB_2101886	1:1000(WB); 1:100(IF)
GRP94	Cell Signaling Technology	20292T	AB_2722657	1:100(IF)
GLUT2	Millipore	400061	AB_211986	1:1000(WB)
Insulin	Cell Signaling Technology	3014S	AB_2126503	1:200(IF)
Glucagon	Cell Signaling Technology	2760S	AB_659831	1:100(IF)
FLAG	Sigma Aldrich	F1804	AB_262044	1:1000(WB); 1:100(IF)
Tubulin	Beyotime	AF1216	AB_2924787	1:1000(WB)
LC3	Cell Signaling Technology	4108S	AB_2137703	1:2000(WB)
BiP(GRP78)	Cell Signaling Technology	3177S	AB_2119845	1:1000(WB)
CHOP	Cell Signaling Technology	2895S	AB_2089254	1:1000(WB)
IRE1α	Cell Signaling Technology	3294S	AB_823545	1:1000(WB)
Phospho- IRE1α	Novus	NB100-2323	AB_3151676	1:1000(WB)
eIF2α	Cell Signaling Technology	9722S	AB_2230924	1:1000(WB)
GAPDH	Beyotime	AF0006	AB_2715590	1:1000(WB)
Alexa Fluor 488-conjugated Goat Anti Rabbit IgG	Invitrogen	461250	AB_2576217	1:100
Alexa Fluor 594-conjugated Goat Anti-Rabbit IgG	Invitrogen	716827	AB_141374	1:100
Alexa Fluor 594-conjugated Goat Anti-Mouse IgG	Invitrogen	443030	AB_141372	1:100
